# Supplementary material for: Response of Fibroblasts from Menkes’ and Wilson’s Copper Metabolism-Related Disorders to Ionizing Radiation: Influence of the Nucleo-Shuttling of the ATM Protein Kinase
Source: Biomolecules. 2023 Dec 5;13(12):1746. doi: 10.3390/biom13121746 (PMC10741441; doi:10.3390/biom13121746)

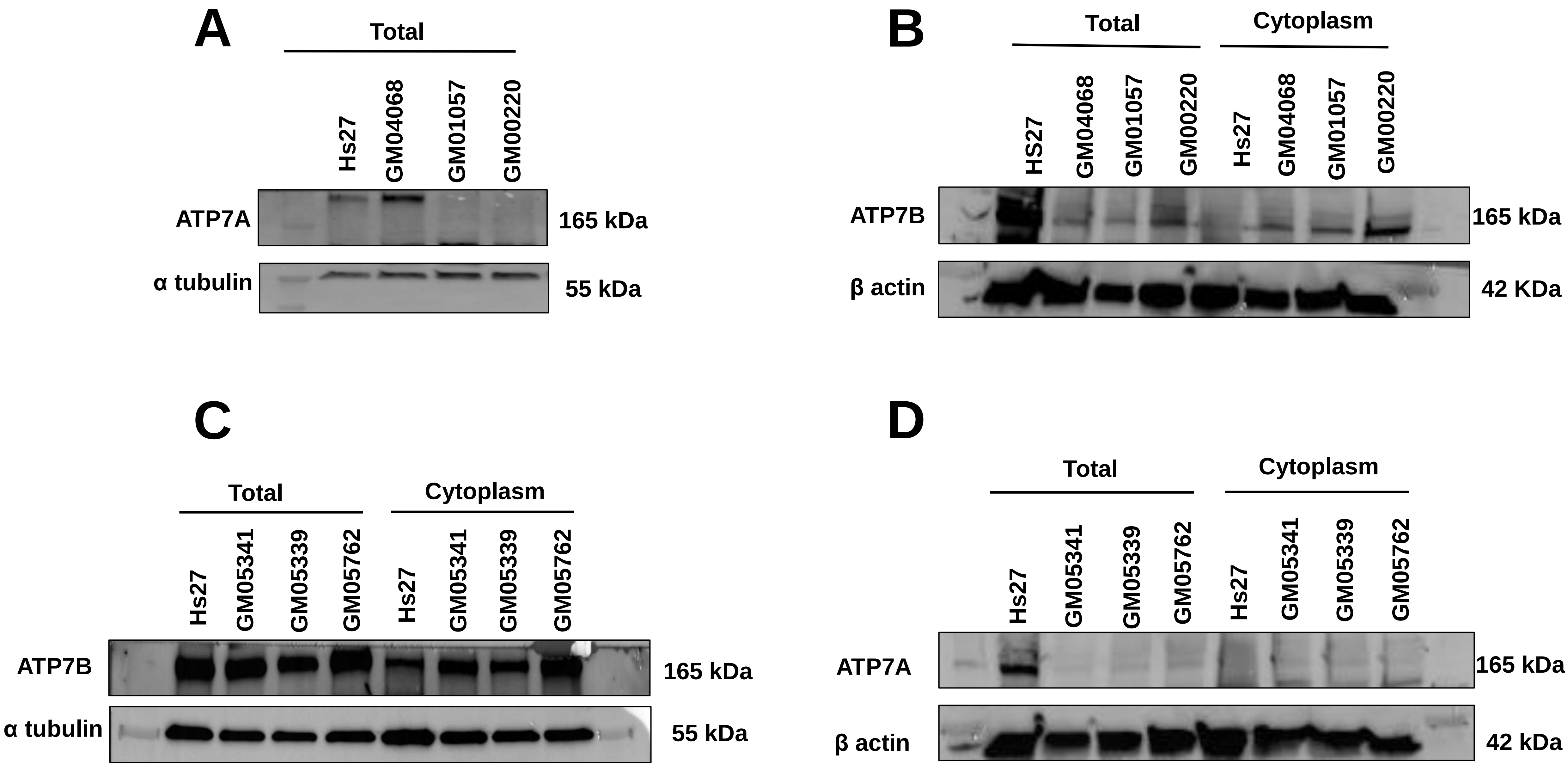
A
Total
Hs27
GM04068
GM01057
GM00220
ATP7A
165 kDa
α tubulin
55 kDa
B
Total
Cytoplasm
HS27
GM04068
GM01057
GM00220
Hs27
GM04068
GM01057
GM00220
ATP7B
165 kDa
β actin
42 KDa
C
Total
Cytoplasm
Hs27
GM05341
GM05339
GM05762
Hs27
GM05341
GM05339
GM05762
ATP7B
165 kDa
α tubulin
55 kDa
D
Total
Cytoplasm
Hs27
GM05341
GM05339
GM05762
Hs27
GM05341
GM05339
GM05762
ATP7A
165 kDa
β actin
42 kDa

Replicat : 1
Cellule : Wilson
Condition: NT
Manip: totale/Cyto-Nuc
Gel: 25/02/2021
Révélation: 26/02/2021

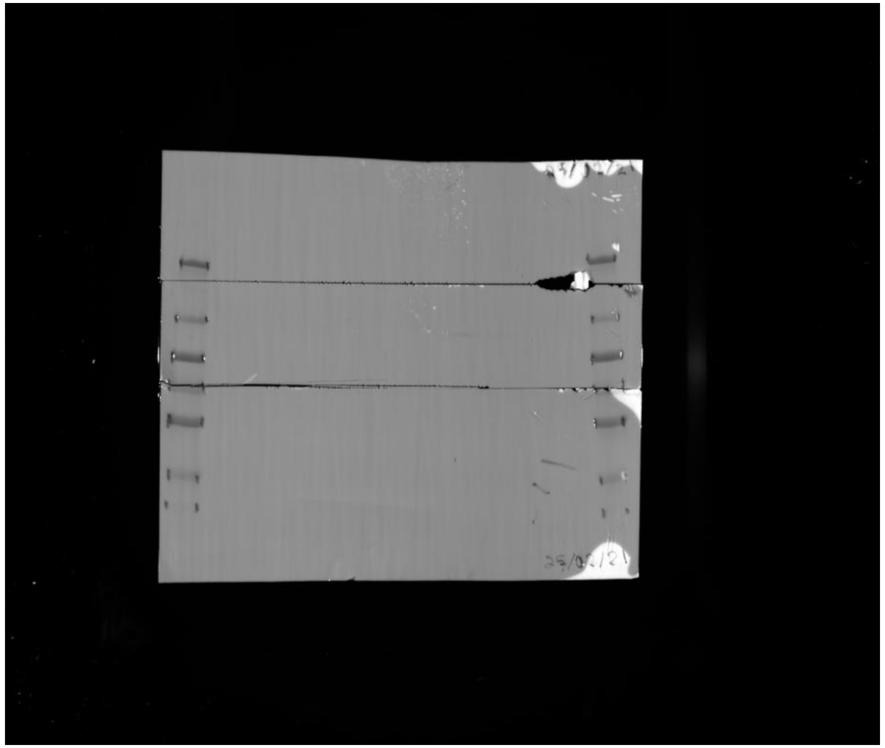

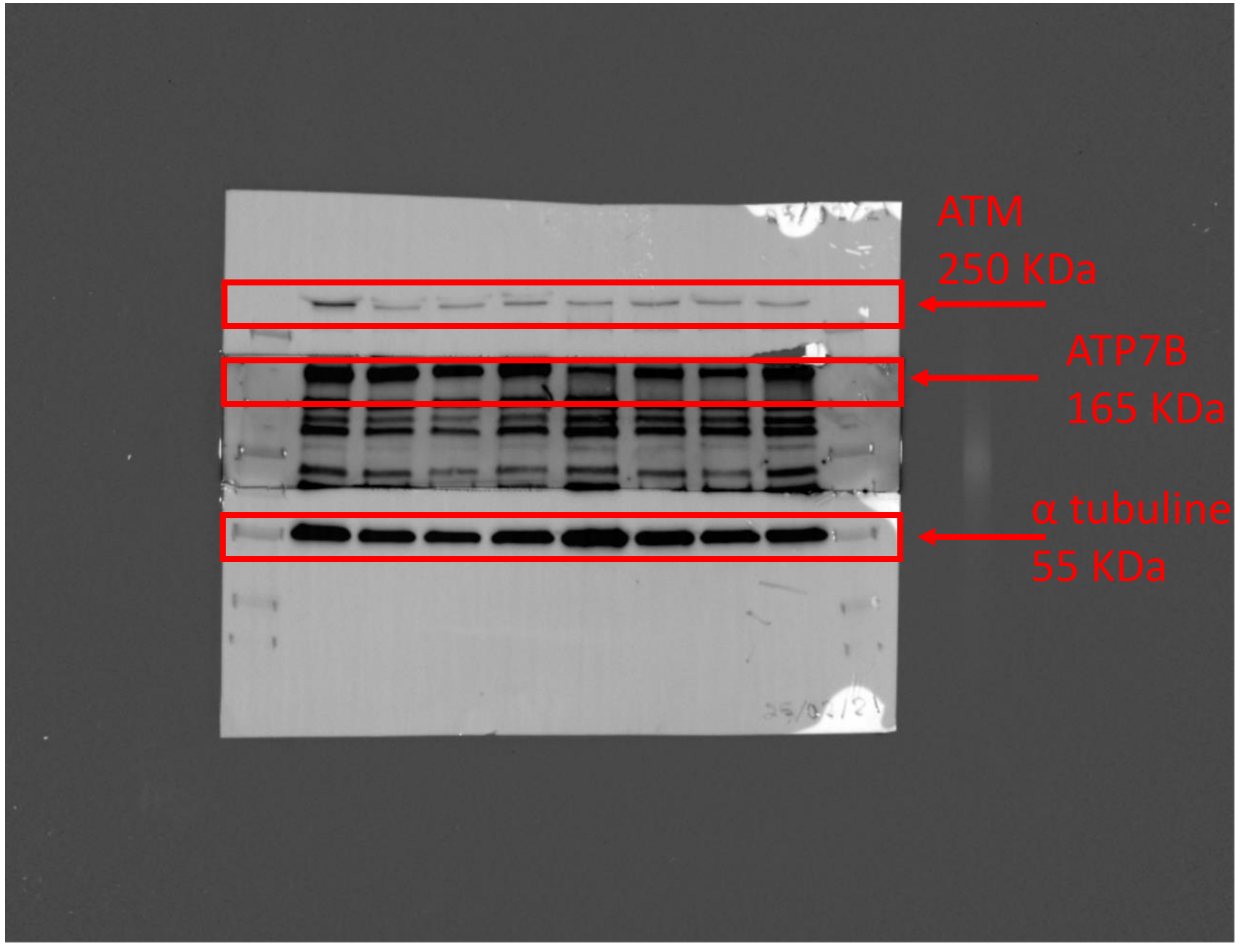

Replicat : 4
Cellule : Menkes
Condition: NT
Manip: total
Gel: 07/05/2021
Révélation: 12/05/2021

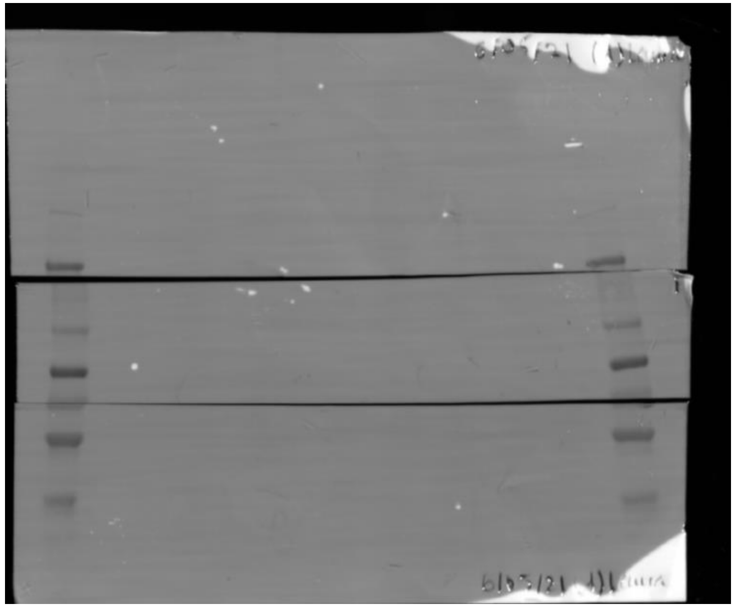

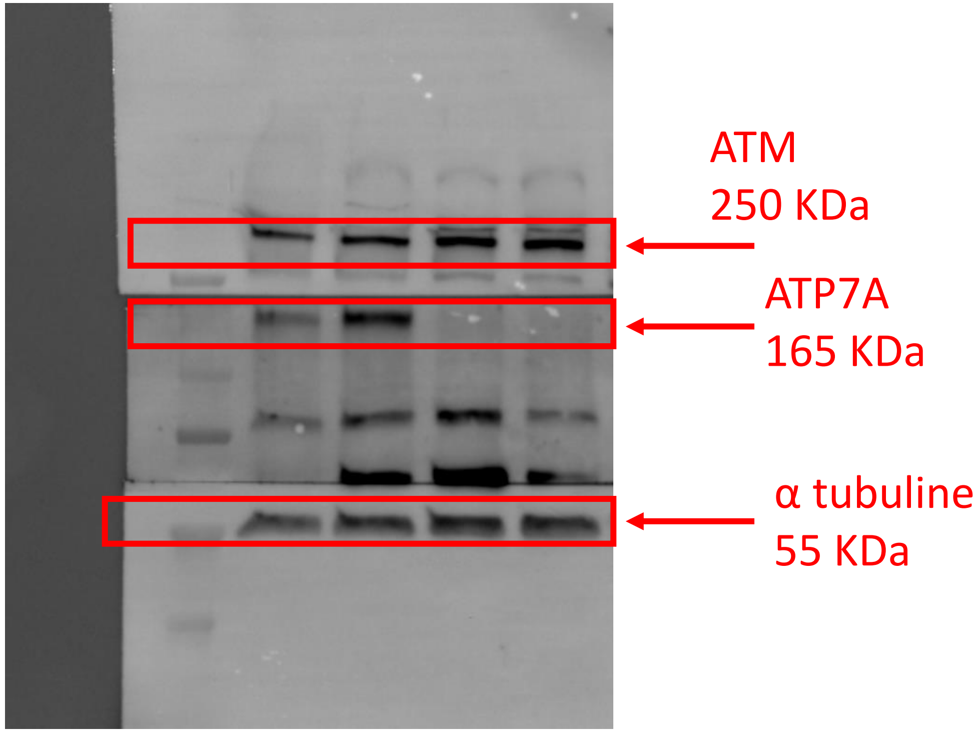

Replicat : 3
Cellules : Wilson & Menkes
Condition: NT
Manip: total/ Cytoplasm
Gel: 23/08/2023
Révélation:24/08/2023

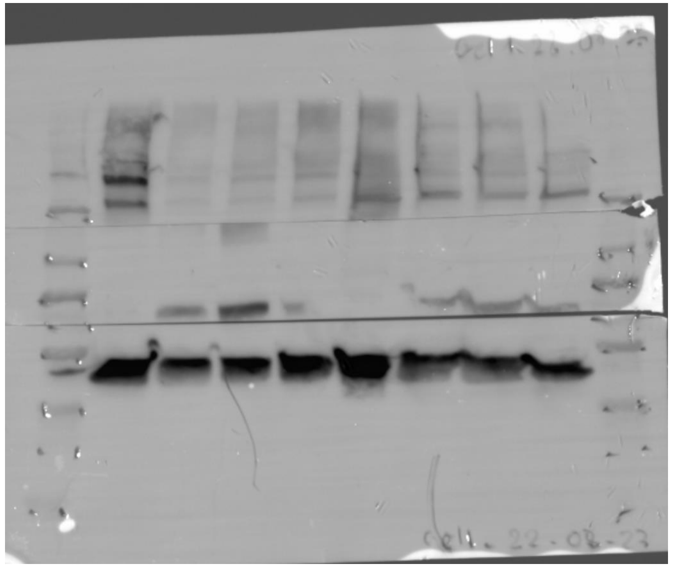

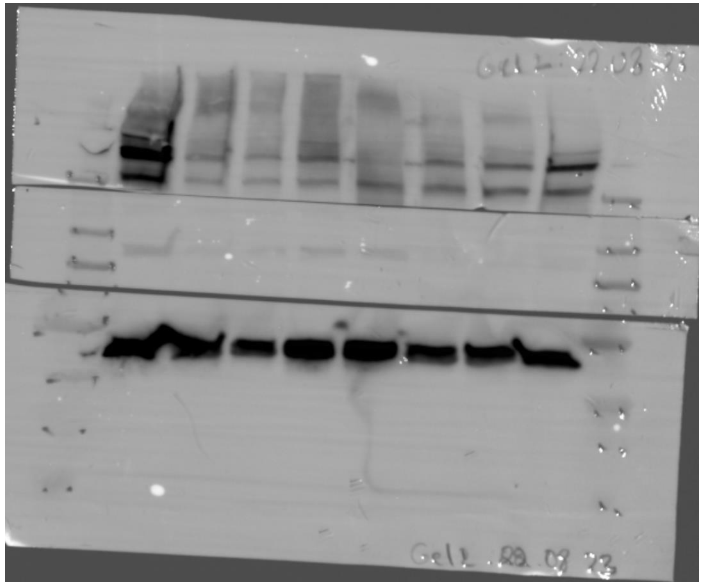

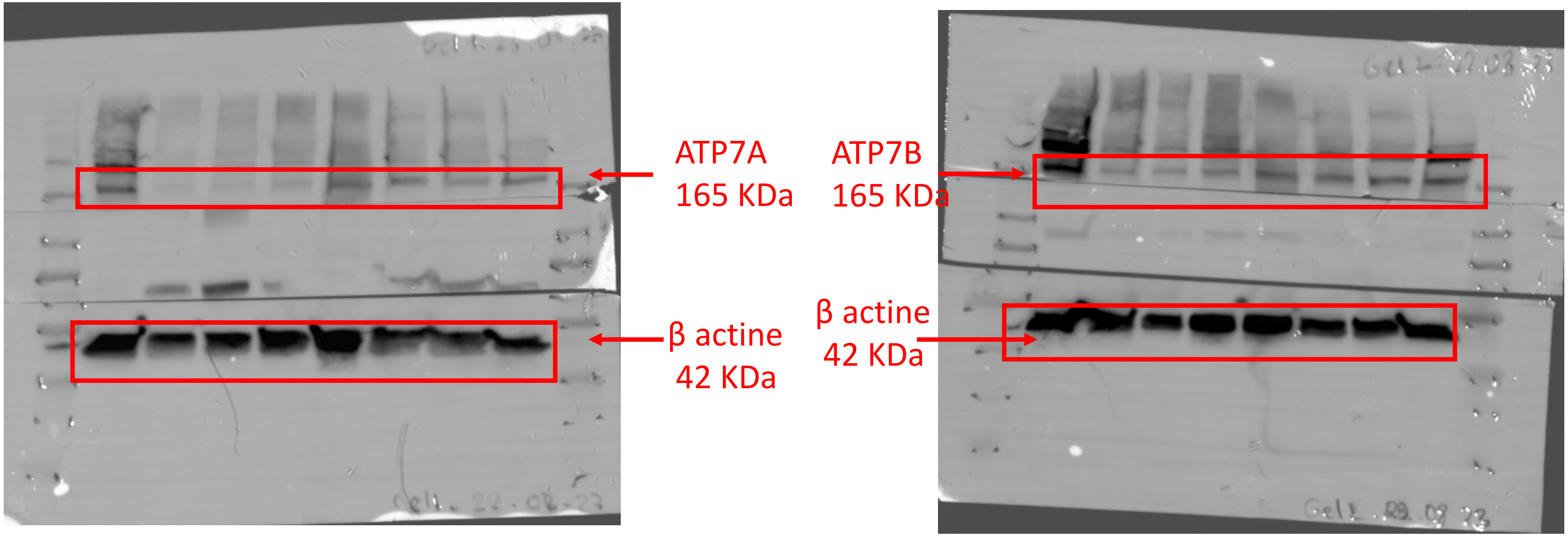

Supplement: Supplementary file 1 [file biomolecules-13-01746-s001.zip › supplementary and WB REVISION - for publish/biomolecules-2699597-raw data.pdf]
