# Supplementary material for: Response of Fibroblasts from Menkes’ and Wilson’s Copper Metabolism-Related Disorders to Ionizing Radiation: Influence of the Nucleo-Shuttling of the ATM Protein Kinase
Source: Biomolecules. 2023 Dec 5;13(12):1746. doi: 10.3390/biom13121746 (PMC10741441; doi:10.3390/biom13121746)
Supplement: Supplementary file 1 [file biomolecules-13-01746-s001.zip › supplementary and WB REVISION - for publish/biomolecules-2699597-supplementary.pdf]

Response of fibroblasts from Menkes and Wilson copper metabolism-related disorders to ionizing radiation: Influence of the nucleo-shuttling of the ATM protein kinase

Supplementary Data

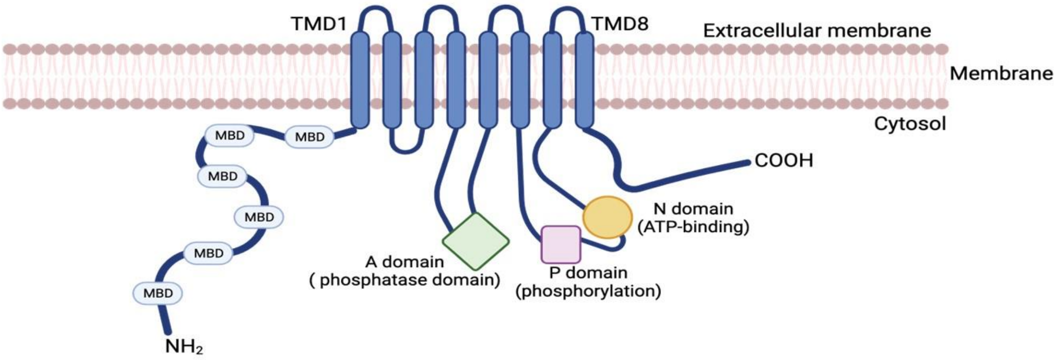

Figure S1 : ATP7A/B structure: both proteins have 8 transmembrane (TMD) domains forming a pore for copper transport across the membrane. ATP7A/B have ATP binding (yellow), phosphatase (green) and phosphorylation (purple) domains, that regulate catalytic activity of the proteins. N-terminal end contains 6 metal binding domains (MBD, blue cylinders) that interact with Cu and regulate protein conformation. The CPC motif in the 6th TMD have a role in the translocation of copper along the channel.

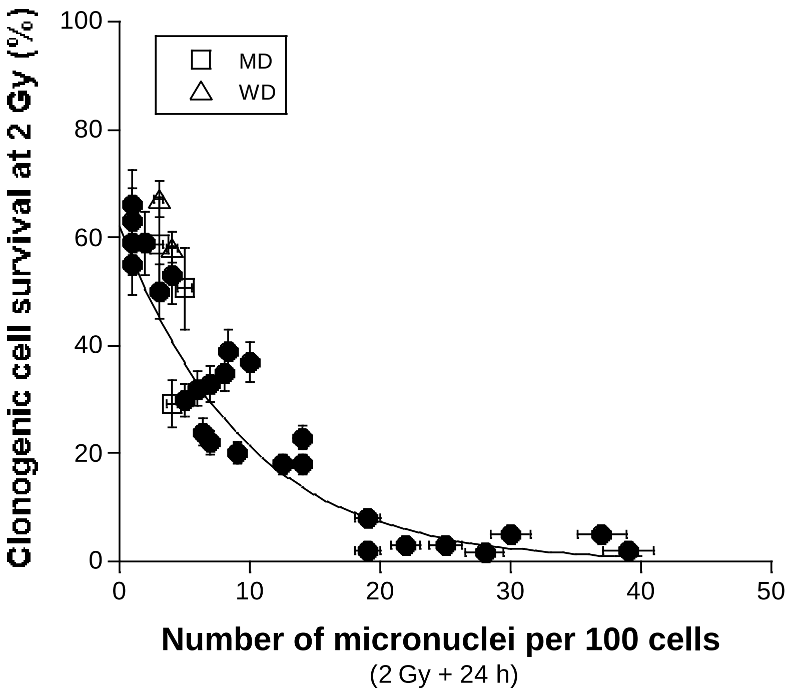

Figure S2 : Relationship between cellular radiosensitivity and residual micronuclei. Survival fraction at 2 Gy (SF2) data were plotted against the corresponding number of micronuclei per 100 cells assessed after 2 Gy X-rays following by 24 h post-irradiation time with cell lines data from the COPERNIC collection (closed symbols) [36]. The MD and WD fibroblasts data obtained in this study were added to the data pool (open symbols). Each plot represents the mean ± standard error of the mean (MEM) of at least three replicates. Data fit corresponds to the following formula : y= 58.57*exp(-0.07x) (r=0.95).

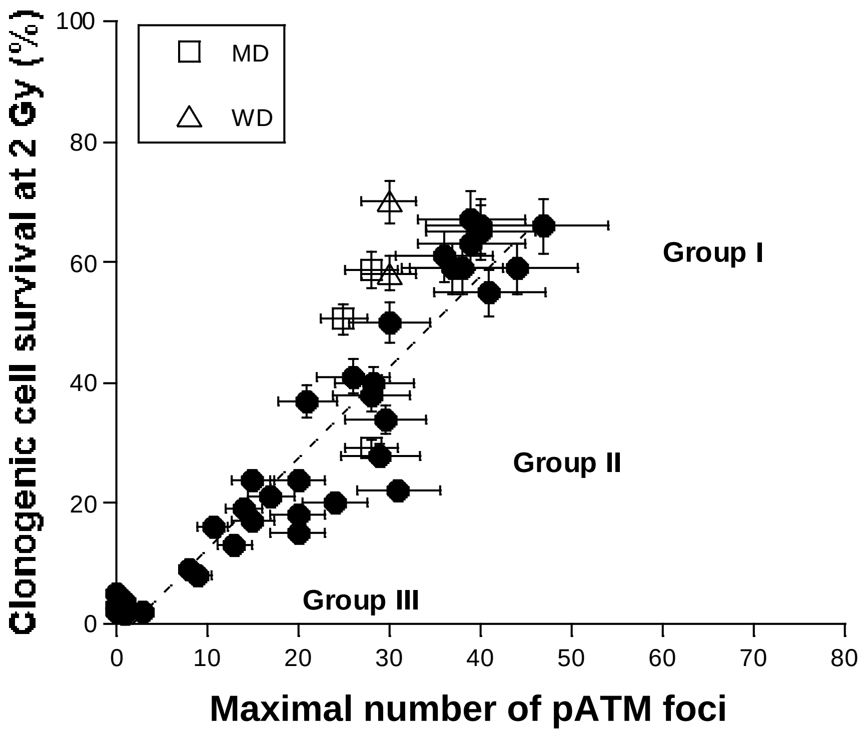

Figure S3 : Relationship between cellular radiosensitivity and the maximal number of pATM foci. Survival fraction at 2 Gy (SF2) data were plotted against the corresponding maximal number of pATM foci cells assessed after 2 Gy X-rays following by 10 min or 1 h post-irradiation time with cell lines data from the COPERNIC collection (closed symbols) [36]. The MD and WD fibroblasts data obtained in this study were added to the data pool (open symbols). Each plot represents the mean ± standard error of the mean (MEM) of at least three replicates. Data fit corresponds to the following formula : y= 1.422*x (r=0.87).

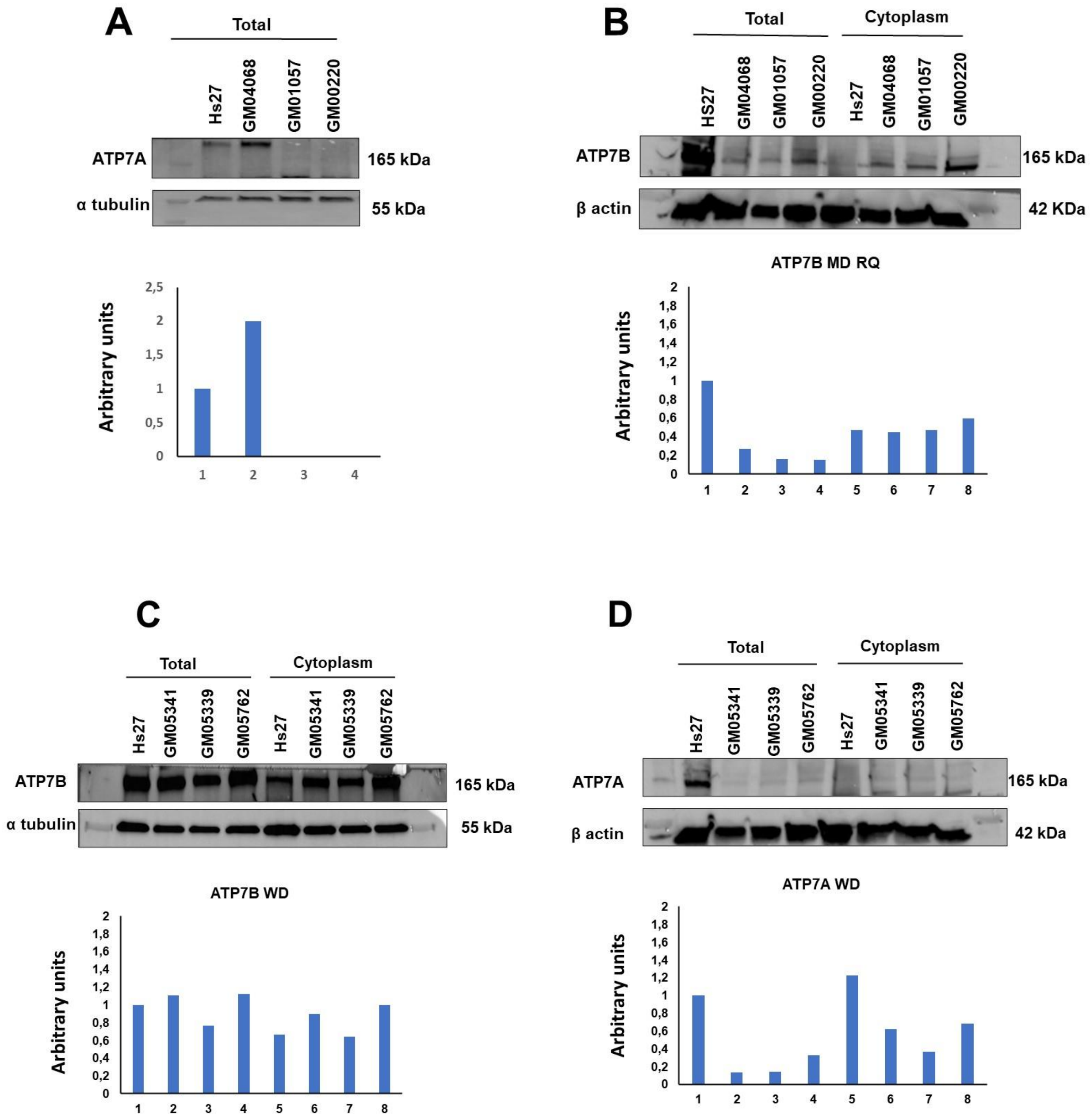

Figure S4. Quantified expression of the ATP7A and ATP7B proteins in MD and WD fibroblasts. Anti-*ATP7A* and anti-*ATP7B* immunoblots with total or cytoplasmic protein extracts were applied to the indicated non-irradiated control Hs27, MD (A, B) and WD (C, D) fibroblasts. The grey levels corresponding to each condition are shown in Fig. S4.

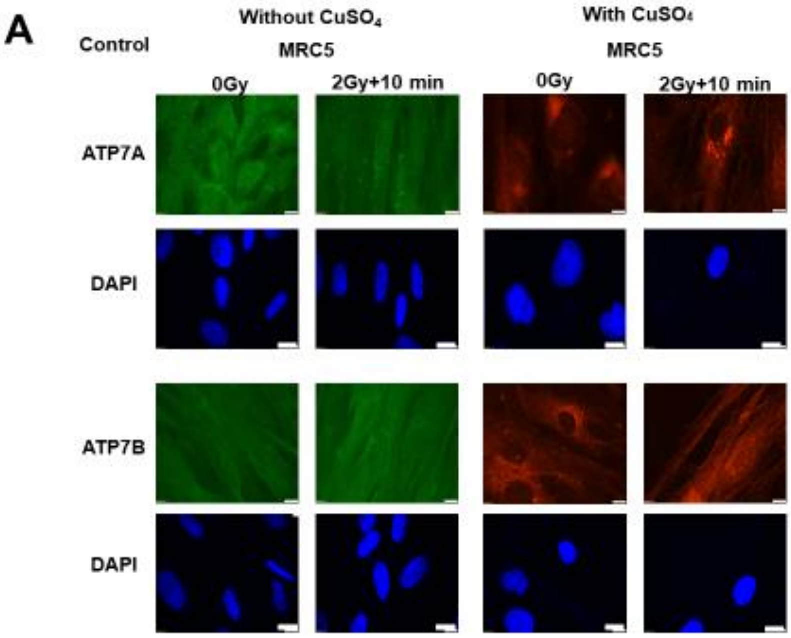
A
Control
Without CuSO$_4$
MRC5
With CuSO$_4$
MRC5
0Gy
2Gy+10 min
0Gy
2Gy+10 min
ATP7A
DAPI
ATP7B
DAPI

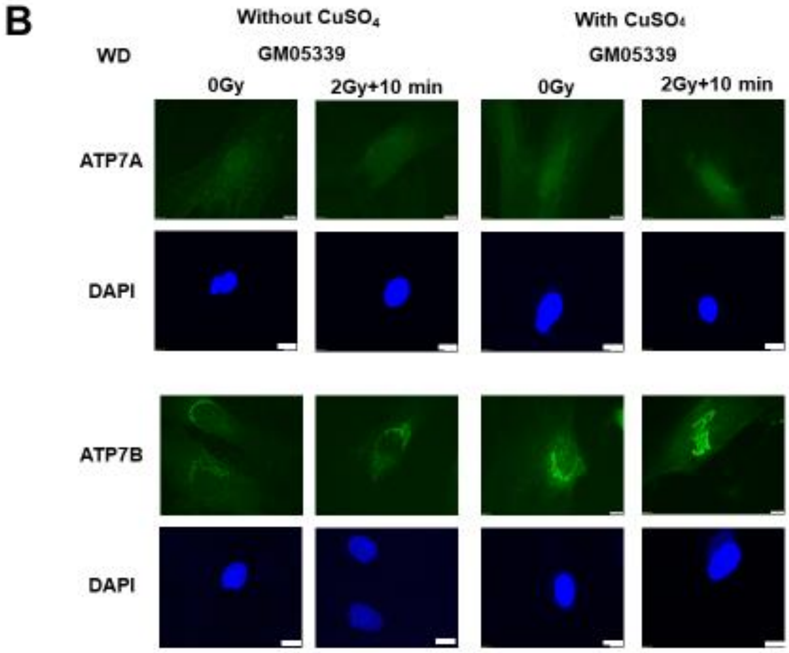
B
WD
Without CuSO$_4$
GM05339
With CuSO$_4$
GM05339
0Gy
2Gy+10 min
0Gy
2Gy+10 min
ATP7A
DAPI
ATP7B
DAPI

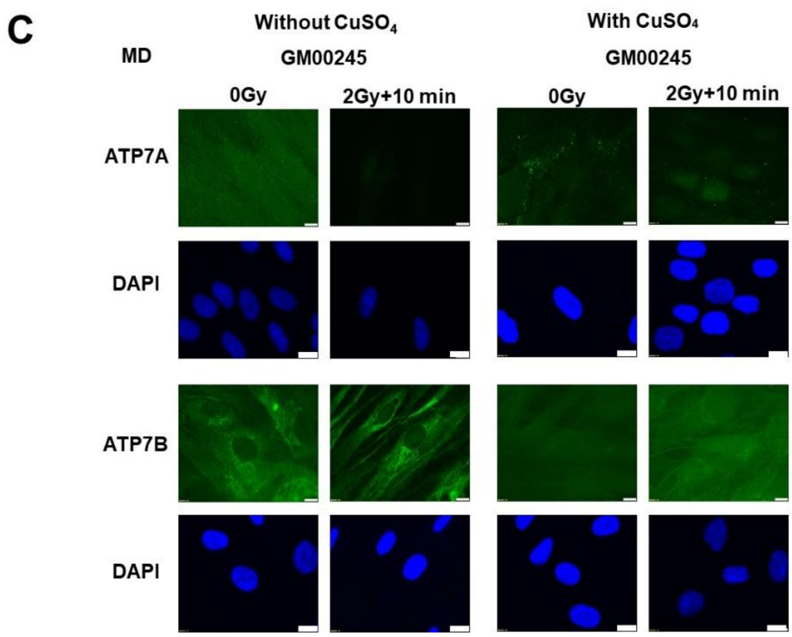

Figure S5 : Sublocalization of ATP7A and ATP7B proteins in MD and WD cells. Representative images taken by immunofluorescence with ATP7A and ATP7B proteins in control (MRC5, A), MD (GM05339, B) and WD (GM00245, C) cell lines at the indicated conditions with or without Cu pre-treatment. Nuclei are counterstained by DAPI. White bars represent 10 μm.

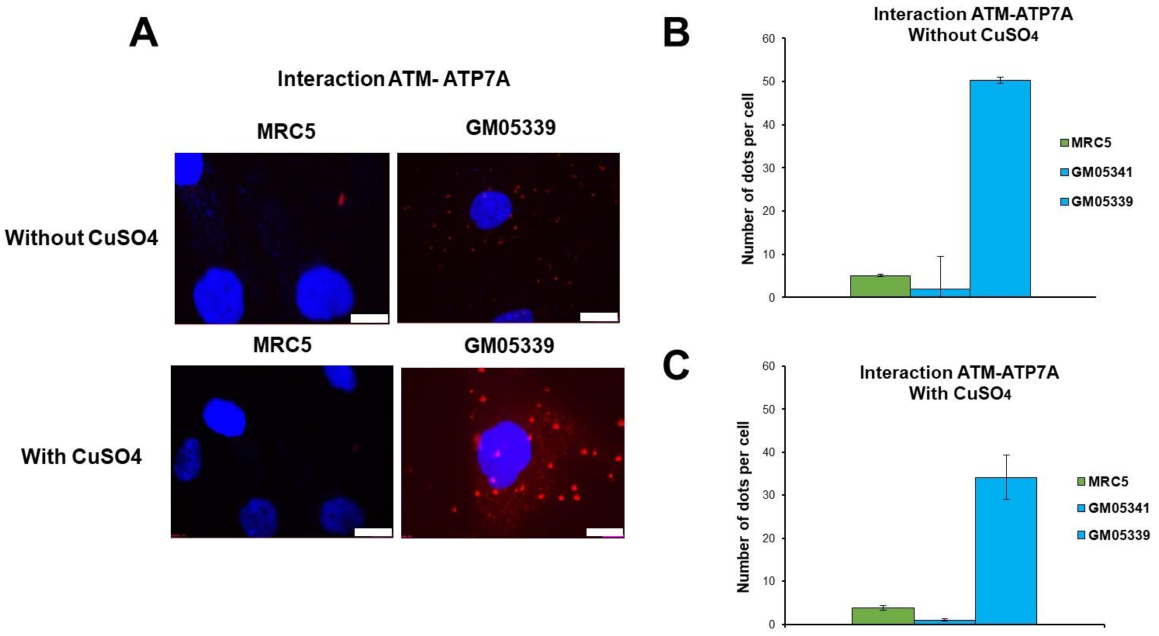

Figure S6. Interaction between ATM and ATP7A observed by applying the proximity ligation assay (PLA) in WD cells. The PLA was applied to the indicated WD cell lines that were exposed or not to Cu-pre-treatment for 24 h. Representative PLA images of ATM-ATP7A complexes observed in the indicated WD cells. The nuclei were counterstained with DAPI (blue). The white bar corresponds to 10 μm. The red foci indicate an ATM-ATP7A protein complex. The white bar corresponds to 10 μm. The average numbers of red foci were scored per 100 cells without (A) or with (B) a Cu-pretreatment Each data point represents the mean ± SEM of two independent replicates.

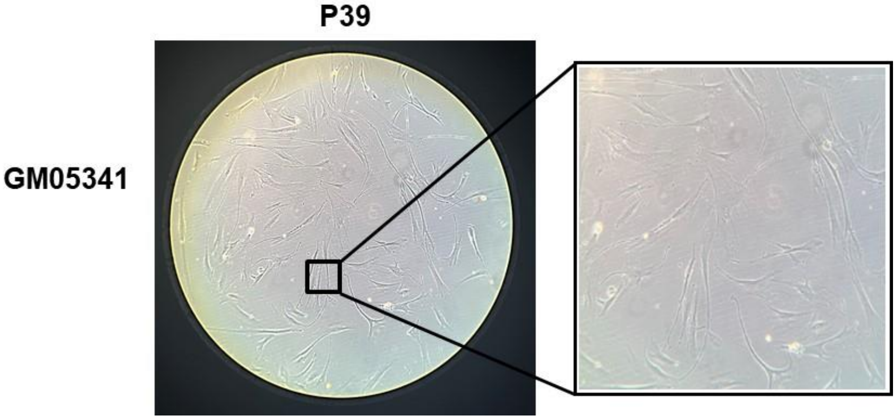

Figure S7 : Representative images of WD cells at culture passages 28-31. Cells routinely cultured in Petri dishes were observed on an inversed microscope cells at the indicated passage. The black bars represent 100 μm.
